# Transdiaphragmatic Intercostal Hernia-An Unusual Hepatic Injury After a Car Accident: A Case Report and Review of the Literature

**DOI:** 10.15190/d.2021.2

**Published:** 2021-03-04

**Authors:** Orestis Ioannidis, Chrysovalantis Mariorakis, Anastasia Malliora, Panagiotis Christidis, Lydia Loutzidou, Ioannis Mantzoros, Manousos George Pramateftakis, Efstathios Kotidis, Nikolaos Ouzounidis, Vasilis Foutsitzis, Stamatios Aggelopoulos

**Affiliations:** 4th Academic Department of Surgery, School of Medicine, Faculty of Health Sciences, Aristotle University of Thessaloniki, Greece

**Keywords:** Hernia, transdiaphragmatic, intercostal, hepatic injury, flail chest.

## Abstract

Transdiaphragmatic intercostal hernia, in which the abdominal contents of the hernia protrude through the diaphragm and the thoracic wall defect. is a very rare type of hernia with only a few cases having been reported in the literature. That type of hernia is usually manifested in male patients after trauma, penetrating or blunt. It is frequently presented with a palpable thoracic mass and pain. The indicated treatment is surgery. We present the case of a 60-year-old female admitted to the hospital after a car accident and suffered multiple rib fractures (6th, 7th, 8th right ribs / 7th, 8th, 9th left ribs), as well as flail thorax, hemothorax bilaterally, left subcutaneous emphysema and swelling of soft tissues of the right lateral thoracoabdominal wall. CT scan revealed herniation of hepatic parenchyma and intestinal loops into the thorax. The patient was treated surgically, and his postoperative course was uneventful. We also review the relevant literature concerning this transdiaphragmatic, intercostal hernia and identify 42 cases. Transdiaphragmatic intercostal hernia is a rare condition, usually manifested in male patients after trauma, penetrating or blunt. It is frequently presented with a palpable thoracic mass and pain. The indicated treatment is surgery.

## INTRODUCTION

Transdiaphragmatic intercostal hernia (TDIH), also called intercostal pleuroperitoneal hernia, in which the abdominal contents of the hernia protrude through the diaphragm and the thoracic wall defect^1^, is a very rare hernia with only 42 cases having been reported in the literature^1-33^^1-33^^1-33^. Table 1 presents in detail all of these cases.

**Table 1:** Reported transdiaphragmatic intercostal hernias in the literature M: male; F: female; R: right; L: left; BP: blood pressure; COPD: chronic obstructive pulmonary disease; GSW: gunshot wound; MVC: motor vehicle crash; VCE: violent coughing episode; subcut.: subcutaneous.

Age/ Gender/Side	Mechanism	Time of Diagnosis	Symptoms	Clinical Presen-tation	Adjoining rib fractures	Level inter-space	Hernia content	Treatment
73/M/L^1^	MVC	6 months	-	Hypotensive, tachycardia	6th to 10th	9th-10th	Small bowel	Surgical repair with polypropylene sutures
82/M/R^1^	Previous surgery	1 year	Pain	Bulge	-	-	Gallbladder fundus	-
19/M/R^2^	GSW	4 months	-	-	8th - 10th	9th	Liver	-
26/M/R^2^	Penetrating	9 months	-	-	6th-10th	9th	Liver	-
27/M/R^2^	GSW	10 months	-	-	9th to 10th	9th	Liver	-
30/M/L^2^	Penetrating	1 year, 3 weeks	-	-	8th -9th	9th	Omentum	-
30/M/L^3^	Blunt Trauma	Immediate	-	-	9th -10th	9th	Omentum	-
58/M//L^4^	Massage	5 months	-	-	None	9th	Empty sac	-
74/M/R^5^	VCE, COPD	3 months	-	-	5th	9th	Empty sac	Surgical repair with a strip of Marlex mesh
57/M/L^6^	Penetrating	None	-	-	None	9th	Omentum	-
72/M/L^7^	VCE, COPD	3 months	Coughing, chest pain	Soft variable mass, ecchymosis	8th	8th	Small bowel	Surgical repair
64/M/L^8^	VCE, pulmonary sarcoidosis	Few weeks	-	-	9th	9th-10th	Small bowel, infarcted omentum	-
69/M/L^9^	MVC	Immediate	Mass	-	7th to 8th	7th- 8th	Bowel	Surgical repair
63/F/L^10^	Coughing	Immediate	Dyspnea, cough, pleurisy, fever,	Wheezing, tachypnea, tachycardia high BP	None	Not stated	Stomach, colon	Surgical repair
23/M/L^11^	Stab wound	2 years	Pain	Bulge, hemithorax	None	9th	Omentum and colon	Thoracotomy
45/M/R^12^	Penetrating (bull gore)	2 years	Swelling after 3 months	Bulge	None	9th	Omentum	Surgical repair with Marlex mesh
73/M/R^13^	Previous surgery, COPD	Immediate	-	-	None (partial resection of the 11th rib)	10th	Small bowel and coecum	Laparotomy, ileocecal resection with ileostomy, polyglactin sutures (Vicryl)
74/M/L^14^	COPD	2 days	Dyspnea on exercise, nausea, vomiting, abdominal pain	Mass	9 – 10th	8th-9th	Μesentery and small bowel	Thoracotomy
74/M/L^15^	Fall – down injury	4 months	Respiratory distress, abdominal pain	Occasional constipation, decreased breathing sound, mass	-	8th	Mesentery	Thoracotomy
77/M/R^16^	MVC	Unknown	Swelling	None	-	7-9th	Colon	Surgical repair either by abdominal or thoracoabdominal approach.
M/L^17^	Spontaneous	5 months	Swelling, breathlessness	Bulge	7th -8th (old)	7th-8th	Small bowel	Thoracoabdominal approach and prolene mesh
69/M/R^18^	Spotaneus	1 year and 9 months	Cough, pain	Hematoma, non-painful mass (after 3 months)	8th-12th	8th-12th	Small intestine loops	Surgical repair with polypropylene mesh
41/M/L^19^	MVC	Immediate	Dyspnea	-	6-9th	-	Spenic and renal	Emergency operation
78/M/L^20^	Spontaneous	Immediate	Pain, dry - paroxysmal cough, inability to pass gas or stool, dark-colored vomiting	Bulge, ecchymosis, atelectasis	-	7th	Colon	Laparoscopy, polypropylene and expanded polytetrafluoroethylene (ePTFE) double mesh
59/F/R^21^	MVC	Immediate	None	Multiple contusions and abrasions, ecchymosis	7th-8th	7th	Transverse colon and splenic flexure	Exploratory laparotomy, middle abdominal incision from xiphoid to pubis
61/M/R^22^	Fall	Immediate	Pain, swelling	Hemopneumothorax	9th-12th	9th-10th	Liver	Laparoscopy, Polypropylene and ePTFE mesh
66/F/R^23^	Fall	2 years	Pain	Bulge	-	9th-10th	Liver and right colonic herniation	Surgical repair, polypropylene mesh
83/F/R^23^	Fall	6 months	Pain, cough, swelling	Hematoma	-	9th-10th	Liver and right colon	Surgical Repair, polypropylene mesh
85/F/L^24^	MVC	Immediate	Pain, acute respiratory distress, swelling	Hypotension, tachycardia	7th-10th	9th-10th	Intra-abdominal contents	Surgical repair
59/M/R^25^	Prior trauma	Unknown	Pain, cough	-	9th	9th	Large bowel	Thoracotomy, prolene mesh
53/M/R^26^	Coughing	1 year	Pain, gastric fullness,	Bulge, hematoma	8th	8th	Part of the colon and omentum	Thoracotomy, prosthetic mesh
71/M/R^26^	Coughing	2 years	Cough, pain	Bulge, ecchymosis	8th	8th	Liver	Thoracotomy
64/M/R^26^	COPD	4 months	Pain, dyspnea, swelling, weakness	Tachypnea, decreased breath sounds, ecchymosis	Last ribs	Last ribs	Abdominal content	Thoracotomy, polypropylene mesh
73/M/L^27^	Coughing	2 years	Pain radiating to the left side of his back	Ecchymosis, nontender mass intermittently	-	7th-8th	Bowel	Surgical repair (transthoracic and abdominal exposure)
38/M/L^28^	Stab injury	2 years	Abdominal pain, dyspnea by two years ago	Hemithorax	-	7th	Transverse colon, omentum and small bowel	ThoracotomyC-core dual mesh
53/F/R^29^	COPD, MVC	6 months	Dyspnea	Mass, Paradox movement (right), hemithorax	8-9th	8th-9th	Segment of Liver, part of the ascending colon along with mesocolon	Thoracotomy, prosthetic patch (Gore Tex)
67/M/L^30^	COPD	2 years	Cough	Ecchymosis, decreased breath sounds (left)	8th	7th-8th	Omentum, splenic flexure, stomach, and anterior spleen	Posterolateral thoracotomy
60/M/R^31^	Coughing	Unknown	Debilitation, dyspnea, cough	Ecchymosis	-	-	Colon, Small bowel and part of the stomach	Thoracotomy with a Dualmesh patch
64/M/L^32^	Coughing	3 days	Flank pain, dyspnea, cough, fever	Hematoma	8th-9th	8th	Bowel	Surgical repair with Polytetrafluoroethylene pledgets
23/M/L^33^	GSW two years ago	Immediate	Vomit, hiccoughs	Left lower lung collapse	-	-	Stomach, Bowel loops	Laparoscopic
69/M/L^34^	None	2 weeks	Abdominal pain, soft swell	Bulge, decreased breath sound, left hemithorax, lower lobe atelectasis	-	-	Stomach, Bowel	Laparotomy with biological mesh
60/F/R (current study)	Car Accident	Immediate	Dyspnea, chest and abdominal pain	Mass, tachypnea, hypotension, subcut. emphysema, bilateral low chest intensive sensitivity, reduced breath sounds, ecchymosis (right), hemithorax	6th-8th right and 7th-9th left	6th- 8th	Liver and bowel	Surgical repair right side pleurodesis, Redon type vacuum

These hernias develop following the disruption and separation of intercostal and diaphragmatic muscles and are almost always acquired following trauma, penetrating or direct blunt, but can seldom present spontaneously^1,17^.

The most common clinical presentation is that of a palpable mass in the chest wall^17^. As with every hernia, a delayed recognition of this injury can prove life-threating, as TDIH can lead to obstruction or strangulation of the intestines^34^.

In the current case report we present a female patient with blunt thoracoabdominal trauma which lead to the development of a right transdiaphragmatic intercostal hernia with the herniation of the right liver lobe through the diaphragm and the fractured 6^th^, 7^th^ and 8^th^ right ribs. A written informed consent was obtained from the patient and the institutional review board of our hospital approved this report. We also review the relevant literature concerning this rare type of hernia. The authors have read the CARE Checklist (2016), and the manuscript was prepared and revised according to the CARE Checklist (2016)^35^.

## CASE REPORT

A 60-year-old female visited the Emergency Department after a car accident, in which she was involved as a co-driver in a side collision with another car. On admission, a**patent airway and severe respiratory distress were noted. Blood pressure (BP) was 100/40 mmHg and heart rate 89/min. The level of consciousness was normal (Glasgow coma scale score 15). Loss of consciousness or peritraumatic amnesia was not mentioned. During transferring, oxygen and intravenous fluids, both in the form of crystalline and colloidal solutions were given. The patient experienced severe chest pain in both hemithoraxes, as well as in the abdomen, especially in the right hypochondrium. The physical examination revealed; paleness, shortness of breath, tachypnea, left subcutaneous emphysema, bilateral low chest tenderness to palpation, flail chest, reduced breath sounds on both lung bases and dullness to percussion. In the right thoracoabdominal wall a palpable painful mass with an ecchymosis of the overlying skin was palpated, while muscular guarding was detected in the abdomen and especially in the right hypochondrium.**She had a known history of coronary heart disease, coronary angioplasty 2 months ago, as well as congenital left hip dislocation.

A central venous catheter in the right internal jugular vein was placed and intravenous fluids and antibiotics (2nd generation cephalosporin) were administered, while a Foley catheter was placed. Given her hemodynamically stability, the patent underwent radiological examination. Chest X-rays revealed fractures of the left clavicle and ribs (6^th^, 7^th^, 8^th^ right ribs / 7^th^,8^th^, 9^th^ left ribs), as well as flail thorax, hemothorax bilaterally, left subcutaneous emphysema and swelling of soft tissues of the right lateral thoracoabdominal wall. Other radiographs showed no pathological findings. Upper and lower abdomen ultrasonography revealed round hypoechoic mass with a capsule measuring 3.7 X 3.9 cm between the visceral surface of the liver and the outer edge of the right colic flexure, while next to it there was an hypoechoic non-capsular hematoma on the lateral abdominal wall. Presence of fluid left pararenal space was detected, while no fluid was revealed in Douglas space.

**Figure 1:**
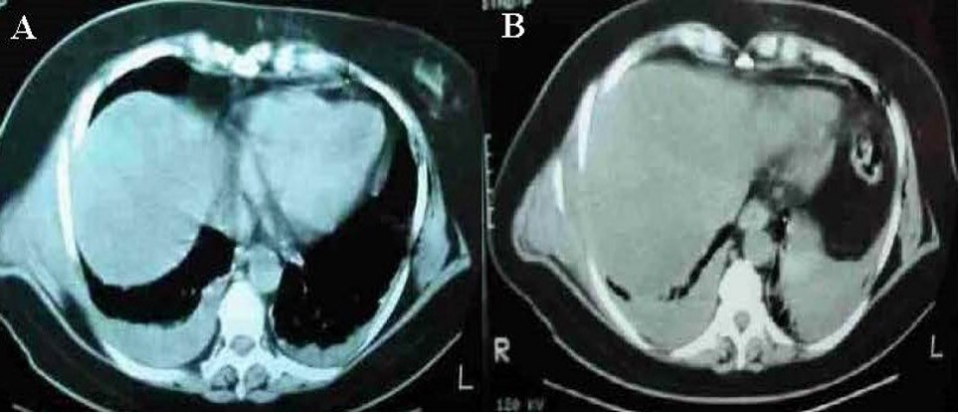
Thoracic CT with bilateral hemothorax Thoracic CT revealed hemothorax bilaterally, but especially on the right, hypoventilation of lower lung fields, left subcutaneous emphysema, rib fractures (6th, 7th, 8th right ribs / 7th, 8th, 9th left ribs), resulting in flail chest. In addition, a large soft tissue hematoma associated to the fractures of the lower thoracic ribs was detected (**A.**) Abdominal CT revealed herniation of hepatic parenchyma through the thoracic wall with concomitant hematoma (**B.**).

Upper and lower abdomen and posterior peritoneum CT reveled hemothorax bilaterally, but especially on the right, hypoventilation of lower lung fields, left subcutaneous emphysema, rib fractures (6^th^, 7^th^, 8^th^ right ribs / 7^th^,8^th^, 9^th^ left ribs), resulting in flail chest. In addition, a large soft tissue hematoma associated to the fractures of the lower thoracic ribs was detected, as well as herniation of hepatic parenchyma and intestinal loops with concomitant hematoma around. A small amount of free intraperitoneal fluid was found perihepatically, left pararenally and in Douglas space, as well as multiple soft tissue injuries of the abdominal wall (Figure 1).

Blood tests showed leukocytosis (white blood cells (WBC) = 12000/mm^3^), anemia (Ht=32.3%), abnormal hepatic function (SGOT=139 mg/dL, SGPT=150 mg/dL) and microscopic hematuria. One hour later hematocrit decreased at 23.2% (hemoglobin = 7.7 g/dL). Electrocardiogram was normal.

Taking all of the above into consideration, the patient underwent surgery immediately. An upper midline incision was conducted in order to examine the abdominal viscera. Two fractured ribs were wedged in the right hepatic lobe, which was emerging between them and towards the soft tissues of the abdominal wall, where there was an extensive hematoma. The right hepatic lobe was restored into the peritoneal cavity, its tears were sutured and a “surgicell” pad was placed over them. A right side pleurodesis was carried out, the diaphragmatic rupture was sutured and drainage of the right subhepatic space was placed. The soft tissue hematoma was accessed and drained and Redon type vacuum was placed. The patient was transfused intraoperatively with two units of blood and she recovered without problems.

The postoperative course was uneventful. Hematocrit gradually increased and transaminases level reduced. Although pleural effusions showed a slight increase during the first days, they gradually declined, without drainage and respiratory function improved. Diuresis was normal during hospitalization, while microscopic hematuria subsided on the 5^th^ postoperative day. On the 13^th^ day of hospitalization, the surgical sutures were removed and on the 14^th^ day a new CT scan revealed a small hernia on the right lateral abdominal wall, with only a small part of hepatic parenchyma protruding. Furthermore, CT scan showed a small right subcapsular hepatic fluid collection, a fluid collection in the fascia of the lateral abdominal wall and bilateral pleural effusion, with accompanying atelectasis of the pulmonary parenchyma. Finally, a subcapsular hematoma of the left kidney was detected, although it was not depicted on the previous CT scan.

On the 17^th^ postoperative day, the patient was discharged due to her good clinical picture and a re-examination after 10 days, including chest radiography and ultrasonography on the upper abdomen was recommended. Two years after this rare thoracoabdominal trauma patient’s course remains uneventful.

## DISCUSSION

The term “Transdiaphragmatic Intercostal Hernia” (TDIH), was first used by Cole et al. in 1986 and describes the herniation of abdominal contents through the diaphragm and the thoracic wall defect^1^and it combines transdiaphragmatic and intercostal hernias, as it results from two simultaneous defects, one affecting the diaphragm while another the intercostal space^17^.We used the PubMed database as the source for a literature review and searched for manuscripts published in English. The search terms used were “transdiaphragmatic intercostal hernia”. The authors reviewed the search results and decided which of these articles should be incorporated into this study. It is a very rare hernia with only 42 cases having been reported in the literature. 83.72% of the patients were males, with ages between 19 and 85 years old, with an average of 57.47^1,2,4-33^.

This hernia develops following the disruption and separation of intercostal and diaphragmatic muscles^1,17^.Although the mechanism of hernia development varies from spontaneous to systematic diseases and traumas, the most common one is trauma, penetrating, or blunt, as in our case, but can seldom present spontaneously^1,2,4-33^. If certain predisposing factors, such as severe asthma and chronic obstructive pulmonary disease, are present, it may be developed after minor incidents, such as coughing^1,36^. It is most commonly associated with rib fractures and especially with 9th or 10th rib fracture^1^. Review of the literature revealed that the level interspace was the 9th in the majority of cases (56.75%) and that hernias were left-sided in 51.16% of the patients^1,2,4-33^. In the present case, the force exerted on the chest by the collision was so great was so massive, that it caused rib fractures and diaphragmatic rupture in the right hemithorax. Τhrough the discontinuity of the diaphragm and due to fractured ribs the majority of right hepatic lobe protruded into the right hemithorax.

Thorough medical history and physical examination may lead to the diagnosis of TDIH. The most common clinical presentation is that of a palpable mass in the chest wall, as in our patient, that fluctuates during the respiratory cycle^1,17^. Thoracic pain, vomiting or dyspnea may also be manifested^1,17^, while on chest auscultation bowel sounds may be heard^37^. Our patient experienced some of these symptoms. Analysis of the patients’ symptoms, found for 30 cases of the literature, and ours, revealed that plethora of symptoms were experienced but mainly pain (67.74%), chest or abdominal, coughing (32.26%) and dyspnea (32.26%), while only one patient (3.23%) was asymptomatic^1,2,4-33^.

Imaging tests can confirm the diagnosis. In particular, chest X-ray can reveal fractures of the ribs, intestinal loops in the thorax and with less sensitivity (17 - 46%) diaphragmatic ruptures^17,38^. Thoracoabdominal ultrasonography and CT scan are the confirmatory imaging tests of choice^17^.CT scan can accurately (61–71% sensitivity, 87–100% specificity) detect diaphragmatic rupture by revealing diaphragm discontinuity, visceral herniation, and collar sign (waist-like intestinal constriction)^38^.It can also confirmthe extent and contents of the hernia (omentum, liver, colon, small bowel or even gall bladder in order of frequency)^36,39^. In our case, although chest X-ray wasn’t able to detect the diaphragmatic rupture nor intestinal content in the thorax, CT revealed the presence of the hernia as well as its content (liver and intestines). Review of the 43 cases, including ours, showed that hernia contained intestines (small or large bowel) in the vast majority of cases and liver was herniating in our patient as well as in 8 more cases (20.93%)^1,2,4-33^. The presentation and thus the diagnosis of TDIH has been reported to be delayed in some cases even for years^1,2,4-33^.A delayed recognition of this injury can prove life-threating, as TDIH can lead to obstruction or strangulation of the intestines^35^.

The treatment of choice for TDIH is surgery, as hernia is usually symptomatic and cannot be reversed automatically due to the negative intrathoracic pressure that tends to enlarge it^15,17^.

Immediate surgery was chosen to be performed in our case as well. The thoracic wall should be first repaired so that the diaphragm can then be fixed at stable attachment points. Large thoracoabdominal incisions that used to be performed in the past tend to be replaced by new techniques, due to the great postoperative pain and the slow mobilization. Nowadays, laparoscopic and endoscopic surgery are increasingly being used to repair uncomplicated intercostal hernias^15^. Video-assisted thoracoscopic surgery could also be performed^39^.Review of the relevant literature reveled that surgical repair was chosen for the treatment in all cases. According to the available data thoracotomy was performed in 11 and laparotomy in 4 cases, while thoracoabdominal approach was chosen in 2 cases. Finally, in 3 more recent cases of 2011, 2012 and 2019 laparoscopic surgery was chosen to be performed^1,2,4-33^.

## CONCLUSION

Transdiaphragmatic intercostal hernia is a rare condition, that has been most commonly reported in male patients after trauma, penetrating or blunt. It is frequently manifested with a palpable thoracic mass and pain and can be diagnosed with CT scan in the majotiry of cases. However, sometimes its presentation and thus its diagnosis may be delayed even for years. The indicated treatment is surgery, which nowadays tends to become less invasive through the use of laparoscopic and endoscopic techniques.

## KEY POINTS

*◊ The most common mechanism of hernia development is trauma, penetrating, or blunt, as in our case, but can seldom present spontaneously. ◊ If certain predisposing factors are present, hernia may be developed after minor incidents, such as coughing. ◊ Transdiaphragmatic Intercostal Hernia” (TDIH) is a very rare hernia with only 42 cases having been reported in the literature. ◊ Review of the literature revealed that in the majority of cases (56.75%) the level interspace was the 9th and that hernias were left-sided in 51.16% of the patients. ◊ The treatment of choice for TDIH is surgical.*
